# Study on the correlation between serum total cholesterol levels and suicidal ideation in patients with first-episode drug-naïve schizophrenia: a cross-sectional study

**DOI:** 10.3389/fpsyt.2025.1701333

**Published:** 2025-11-25

**Authors:** Jiale Wang, Cen Li, Xiaojuan Zhang, Xiaoe Lang

**Affiliations:** Department of Psychiatry, First Hospital of Shanxi Medical University, Taiyuan, China

**Keywords:** schizophrenia, suicidal ideation, total cholesterol, correlation analysis, risk prediction

## Abstract

**Background:**

The pathological physiology of schizophrenia (SZ) is associated with defects in biological energy metabolism. Abnormal blood lipid levels may influence the emergence of suicidal ideation (SI) in SZ. This study aims to evaluate the relationship between blood lipid levels, psychiatric symptoms, and SI in first-episode drug-naïve (FEDN) SZ patients.

**Method:**

This study included 183 patients with FEDN SZ and 101 healthy controls (HC). The Beck Scale for Suicide Ideation Chinese Version and the Positive and Negative Syndrome Scale five-factor model were utilized to assess the intensity of SI and the clinical symptoms of the participants. All subjects underwent testing for levels of total cholesterol (TC), triglycerides (TG), high-density lipoprotein (HDL), and low-density lipoprotein (LDL). Relevant variables were identified through differential comparisons and logistic regression analysis. The risk was evaluated using the area under the Receiver Operating Characteristic (ROC) curve (AUC).

**Result:**

Compared with the HC group, the levels of serum TC, TG and LDL in FEDN SZ patients were higher, while the HDL level decreased (P<0.05). The levels of serum TC, HDL, LDL, positive and depressive symptom factor scores of FEDN SZ patients with SI were higher (P<0.05). Stepwise regression scores showed that serum TC levels, positive and depressive symptom factor scores were positively correlated with the generation of SI in FEDN SZ patients (P<0.05); The AUCROC shows that the combined discriminant efficacy of the three indicators is AUC = 0.824.

**Conclusion:**

The higher the serum TC levels, positive symptom factor scores, and depressive symptom factor scores in FEDN SZ patients, the greater the risk of SI. Therefore, timely measurement of serum TC levels during the initial diagnosis of FEND SZ patients can help promptly initiate therapeutic interventions, reduce the incidence of SI, and improve the prognosis of FEND SZ patients.

## 1 Introduction

Schizophrenia (SZ) is a severe mental illness associated with a high risk of suicide among patients (1). A 21-year follow-up study conducted in rural China revealed that the suicide rate among SZ reached 13.8% (2), with the risk of suicide being 8.5 to 13 times greater than that of the general population (3, 4). Notably, during the early stages of SZ, the risk of suicide increases by 60% compared to later stages (5). Evidence suggests suicidal ideation (SI) is a strong indicator of suicidal behavior. SZ patients with SI have a markedly higher risk of engaging in suicidal behavior in the future compared to those who do not have SI (6). Therefore, investigating the factors associated with SI is of great significance for identifying and reducing the risk of future suicidal behaviors in patients with SZ.

Patients with SZ are at an elevated risk for dyslipidemia, with some studies indicating that approximately 40% of SZ patients exhibit abnormal lipid metabolism (7). There is increasing evidence suggesting that these metabolic abnormalities may manifest in the early stages of the disease, even prior to the initiation of pharmacological treatment (7–10). Research has demonstrated a close association between dyslipidemia and the pathophysiological processes underlying SZ (11, 12). It is hypothesized that lipid metabolic disturbances within brain tissue can influence the synthesis and release of neurotransmitters, thereby impacting cognitive and emotional functioning in individuals with SZ (11). Furthermore, many SZ patients may encounter metabolic disorders, including dyslipidemia, as a consequence of antipsychotic medication, which further exacerbates their overall health risks (10, 13). In addition, some studies have shown that metabolic syndrome and diabetes may lead to neurodegeneration, and both neurodevelopmental and neurodegenerative processes can cause SZ (14), which may be related to abnormal expression of miRNA (15).

Recent studies have demonstrated a significant association between serum total cholesterol (TC) levels and suicide risk in patients with SZ. Low serum TC levels may serve as a biomarker for suicide attempts (16). A Polish study indicated that low levels of TC, triglycerides (TG), low-density lipoprotein (LDL), and total lipids may serve as status-dependent risk factors for suicidal behavior in individuals with schizophrenia (17). Conversely, elevated serum TC levels could influence the neurotransmitter system, the stability of neurocellular membranes, and the inflammatory response, which in turn may impair emotional regulation and impulse control, thereby increasing the risk of suicide (18). Furthermore, psychotic symptoms—including positive symptoms, negative symptoms, depressive symptoms — and drug abuse are correlated with a heightened risk of suicide in SZ patients (19). However, existing studies remain controversial regarding the relationship between blood lipid levels and SI in patients with first-episode drug-naïve (FEDN) SZ.

Recent studies have indicated that patients with SZ are at an increased risk of dyslipidemia when treated with antipsychotic medications (7, 20). Thus, this study enrolled FEDN SZ patients as subjects to investigate the relationship between their dyslipidemia and SI, which can largely avoid the interference of potential confounding factors such as antipsychotic medications and disease duration on the research results. In this context, 183 FEDN SZ patients and 101 healthy controls (HC) were recruited for this study to identify risk factors associated with the emergence of SI in FEDN SZ patients, utilizing both lipid levels and the Positive and Negative Syndrome Scale (PANSS). An innovative multi-dimensional prediction model was developed, integrating lipid levels and the PANSS five-factor model, to pinpoint independent predictors of suicide ideation risk in SZ patients. Furthermore, the predictive efficacy of these factors was assessed through receiver operating characteristic (ROC) curves, highlighting the novelty of this research within the field of SZ.

## 2 Subjects and methods

### 2.1 Subjects

This study adopted a cross-sectional design. The participants included 183 FEDN SZ patients who received inpatient or outpatient treatment at First Hospital of Shanxi Medical University from July 2023 to December 2024. The enrollment criteria include:(1)SZ patients who meet the diagnostic criteria outlined in the Diagnostic and Statistical Manual of Mental Disorders, 5th Edition (DSM-5) (21); (2) aged 13 to 60 years; (3) of Han nationality; (4) first episode and no medication taken before; (5) duration of illness not exceeding 5 years; (6) informed consent obtained from the patient’s legal guardian. The exclusion criteria are as follows:(1) individuals meeting other diagnostic criteria from the DSM-5; (2) suffering from physical diseases; (3) being unable to cooperate with the study due to alcohol abuse, impulsivity, or agitation; (4) having taken lipid-lowering drugs (e.g., statins, fibrates) within the past 2 months or currently.

Additionally, 101 healthy control (HC) participants were recruited, matched with the SZ patient group in terms of gender and age. Two independent psychiatrists assessed these HC participants to evaluate their current mental status and confirm the presence of personal or family history of psychiatric disorders. HC participants were excluded if they had SI or a personal/family history of psychiatric disorders. All HC participants underwent a comprehensive physical examination, and detailed medical history data were collected. HC participants were excluded from the study if they met any of the following criteria: (1) suffering from physical diseases; (2) having taken lipid-lowering drugs (e.g., statins, fibrates) within the past 2 months or currently.

This study received approval from the Ethics Review Committee of First Hospital of Shanxi Medical University, and all subjects provided informed consent.

### 2.2 Research methods

#### 2.2.1 General data collection

This study collected general data from patients, including basic information such as sex, age, marital status, years of education, body mass index (BMI), smoking and alcohol history and Course of disease.

#### 2.2.2 Serum test

After a 12-hour fasting period, 5 mL of blood was drawn from the cubital veins of the subject in the morning. The blood samples were promptly processed following collection and subsequently stored in an ultra-low temperature environment at -80 °C. Serum TC, TG, high-density lipoprotein (HDL), and LDL levels were measured using the AU5800 automated chemical analyzer. The detection method was conducted rigorously by a designated individual in accordance with the specified guidelines. The normal ranges for the serum detection indicators are as follows: TC: 3.25-5.18 mmol/L; TG: 0.2-1.7 mmol/L; HDL: ≥1.04 mmol/L; LDL: 0-3.12 mmol/L.

#### 2.2.3 Scale evaluation

Two psychiatrists, who were consistently trained, utilized the Positive and Negative Syndrome Scale (PANSS) (22) and the Beck Scale for Suicide Ideation - Chinese Version (BSI-CV) to evaluate the psychotic symptoms and suicidal intentions of patients with SZ (1). PANSS scale: In recent years, a five-factor model of the Positive and Negative Syndrome Scale (PANSS) has been proposed based on factor analysis. This model includes positive symptom factors, negative symptom factors, cognitive symptom factors, excitatory symptom factors, and depression symptom factors. When it comes to the PANSS, the positive symptom factor is made up of four key elements: “delusion” (P1), “hallucinations” (P3), “exaggeration” (P5), and “unusual thinking content” (G9). Shifting gears to negative symptoms, we are looking at six PANSS components: “emotional dullness” (N1), “emotional withdrawal” (N2), “emotional communication disorder” (N3), “passive/indifferent social withdrawal” (N4), “lack of spontaneity and fluency in conversation” (N6), and “slow movement” (G7). As for cognitive symptoms, three PANSS indicators come into play: “attention disorder” (G11), “abstract thinking difficulty” (N5), and “poor concentration” (P2). Then there is the excitatory symptom factor, which boils down to four PANSS items: “excitement” (P4), “hostility” (P7), “non-cooperation” (G8), and “lack of impulse control” (G14). Last but not least, the depression symptom factors are rounded out by three more PANSS items: “anxiety” (G2), “self-incrimination” (G3), and “depression” (G6) (2). BSI-CV: In this study, patients with SZ were classified based on their scores from the 4th and 5th items of the BSI-CV. The patients were divided into two groups: those with SI and those without. Scores on item 4, which reflects active SI, or item 5, which indicates passive SI, were categorized as “weak” or “medium to strong” to denote the level of SI. Conversely, if the scores for both items 4 and 5 were recorded as “none,” it indicated that the patient did not exhibit any SI.

### 2.3 Statistical methods

The data were statistically analyzed using R version 4.4.2. Normally distributed metric data were presented as the mean ± standard deviation (X ± s), and we used t-tests to compare the groups. For data that were not normally distributed, we showed the median with the interquartile range [M (Q1, Q3)], and group comparisons were done using the Mann-Whitney U test (also known as the rank sum test). We described categorical variables with frequencies and percentages, and the chi-squared test was our go-to for comparing them across groups. Initially, a descriptive statistical analysis of the patients’ clinical information was performed. Subsequently, continuous variables underwent analysis of variance (ANOVA), while categorical variables were subjected to chi-square tests. Demographic and clinical information were compared, followed by binary logistic regression analysis, where SI served as the dependent variable. The covariates included smoking and alcohol status, duration of the illness, BMI, TC, TG, HDL, LDL, PANSS total scores, and their respective factor scores to identify relevant factors associated with SI in patients with SZ. Finally, the area under the receiver operating characteristic curve (AUC-ROC) was calculated to identify risk factors distinguishing SI in SZ patients. The significance level was set at α = 0.05 for two-sided tests.

## 3 Results

### 3.1 Comparison of the differences in general demographic information and the four levels of blood lipids between SZ patients and HC

There were no statistically significant differences in general demographic data such as sex, age, and years of education between SZ patients and the HC group (P > 0.05). Compared with the HC group, the patients in the FEDN SZ group had higher levels of TC, TG and LDL, and lower levels of HDL, and the differences were statistically significant (P < 0.05) (See **Table 1**).

**Table 1:** Comparison of the differences in general demographic data and the four levels of blood lipids between SZ patients and HC.

Variables	HC (n = 101)	SZ (n = 183)	χ2/Z/t	*P*
Sex [n (%)]			χ²=0.89	0.345
Male	45 (44.55)	71 (38.80)		
Female	56 (55.45)	112 (61.20)		
Age (years)	25.00 (23.00, 30.00)	24.00 (18.00, 34.00)	Z=-1.92	0.055
Education (years)	12.00 (9.00, 15.00)	12.00 (9.00, 15.00)	Z=-1.31	0.192
BMI (kg/m²)	22.49 (21.10, 24.18)	23.12 (21.26, 26.26)	Z=1.64	0.101
Smoking [n (%)]			χ²=0.002	0.965
Yes	18 (17.82)	33 (18.03)		
No	83 (82.18)	150 (81.97)		
Alcohol [n (%)]			χ²=0.66	0.416
Yes	8 (7.92)	10 (5.46)		
No	93 (92.08)	173 (94.54)		
TC (mmol/L)	4.53 (4.03, 5.11)	4.03 (3.52, 4.91)	Z=-3.41	<0.001***
TG (mmol/L)	0.97 (0.74, 1.22)	1.11 (0.83, 1.53)	Z=3.03	0.003**
HDL (mmol/L)	1.29 (1.14, 1.60)	1.25 (1.06, 1.47)	Z=-2.51	0.012*
LDL (mmol/L)	2.80 (2.44, 3.22)	2.51 (2.04, 3.09)	Z=-3.45	<0.001***

Comment: The data are presented as median (interquartile range) [M (P25, P75)]; the bold value indicates *p* < 0.05; *p < 0.05; **p < 0.01; ***p < 0.001.

### 3.2 Comparison of the differences in general demographic information and the four levels of blood lipids among the SZ patients with and without SI

There were no significant differences in general demographic data, including sex, age, and years of education, among patients with SZ (*P* > 0.05). However, when we compared the SZ patients who were having SI to those who were not, the group with SI showed higher levels of TC, HDL, and LDL. Additionally, the SI group showed higher scores on positive symptom factors and depression symptom factors, and the differences were statistically significant (*P* < 0.05). However, the two groups were statistically indistinguishable when it came to TG levels, negative symptom factors, cognitive symptom factors, excitatory symptom factors, and total PANSS scores (*P* > 0.05) (Refer to **Table 2**).

**Table 2:** Comparison of differences in the general information, the levels of 4 blood lipids, and the PANSS scale among the SZ patients with or without SI.

Variables	SZ without SI (n = 101)	SZ with SI (n = 82)	χ2/Z/t	*P*
Sex [n (%)]			χ²=3.15	0.076
Male	45 (44.55)	26 (31.71)		
Female	56 (55.45)	56 (68.29)		
Age (years)	26.00 (18.00, 32.00)	20.00 (16.75, 35.25)	Z=-1.34	0.180
Education (years)	12.00 (9.00, 15.00)	11.00 (9.00, 14.00)	Z=-1.89	0.059
BMI (kg/m²)	22.86 (21.52, 25.56)	24.43 (20.36, 26.91)	Z=0.92	0.358
Course of disease (years)	1.00 (0.29, 2.83)	2.00 (0.42, 3.00)	Z=1.27	0.203
Smoking [n (%)]			χ²=2.14	0.143
Yes	22 (21.78)	11 (13.41)		
No	79 (78.22)	71 (86.59)		
Alcohol [n(%)]			χ²=0.94	0.333
Yes	7 (6.93)	3 (3.66)		
No	94 (93.07)	79 (96.34)		
TC (mmol/L)	3.70 (3.45, 4.18)	4.74 (4.20, 5.21)	Z=4.70	<0.001^***^
TG (mmol/L)	1.06 (0.79, 1.54)	1.17 (0.93, 1.52)	Z=1.33	0.182
HDL (mmol/L)	1.21 (1.01, 1.43)	1.28 (1.10, 1.52)	Z=2.09	0.036^*^
LDL (mmol/L)	2.24 (1.97, 2.72)	2.87 (2.39, 3.31)	Z=4.65	<0.001^***^
PANSS				
Positive symptoms	10.00 (7.00, 14.00)	12.00 (9.75, 18.00)	Z=3.50	<0.001^***^
Negative symptoms	21.00 (16.00, 29.00)	21.00 (16.00, 29.00)	Z=-0.18	0.854
Cognitive symptoms	10.00 (6.00, 15.00)	11.00 (6.00, 15.00)	Z=-0.02	0.983
Excitatory symptoms	8.00 (4.00, 13.00)	10.00 (6.00, 14.25)	Z=1.59	0.112
Depressive symptoms	9.00 (7.00, 11.00)	11.00 (9.00, 14.00)	Z=5.06	<0.001^***^
Total	98.00 (77.50, 120.50)	102.50 (77.00, 120.25)	Z=0.49	0.627

Comment: Data are presented as mean ± standard deviation (X̄ ± s) and median (interquartiles) [M (P25, P75)]. Bold values indicate *p* < 0.05; *p < 0.05; **p < 0.01; ***p < 0.001.

### 3.3 Related factors in the development of SI in patients with FEDN SZ

#### 3.3.1 Univariate, multivariate analysis and stepwise logistic regression analysis

As shown in **Table 3**. In the multivariate logistic regression analysis, whether FEDN SZ patients had SI was taken as the dependent variable, and the statistically significant changes (TC, HDL, LDL, positive symptom factor, depressive symptom factor) in **Table 2** were included as independent variables. The results showed that only TC (*OR* = 7.80, *P* < 0.05) and the depressive symptom factor (*OR* = 1.30, *P* < 0.001) were significantly correlated with whether FEDN SZ patients had SI. When the TC level and the score of the depressive symptom factor were higher, the possibility of SZ patients having SI was higher.

**Table 3:** Univariate and multivariate logistic regression analysis.

Variables	Univariate logistic regression analysis				Multivariate logistic regression analysis			
	*β*	*S. E*	*P*	*OR (95%CI)*	*β*	*S. E*	*P*	*OR (95%CI)*
TC	0.73	0.18	<0.001^***^	2.08 (1.47 ~ 2.96)	2.05	0.90	0.023^*^	7.80 (1.33 ~ 45.66)
HDL	0.95	0.48	0.050	2.58 (1.00 ~ 6.63)	-0.07	1.16	0.952	0.93 (0.10 ~ 8.99)
LDL	0.83	0.23	<0.001^***^	2.29 (1.46 ~ 3.60)	-0.46	1.21	0.700	0.63 (0.06 ~ 6.67)
Positive symptoms	0.10	0.03	<0.001^***^	1.11 (1.04 ~ 1.17)	-0.20	0.11	0.073	0.82 (0.66 ~ 1.02)
Depressive symptoms	0.28	0.06	<0.001^***^	1.33 (1.18 ~ 1.49)	0.26	0.06	<0.001^***^	1.30 (1.16 ~ 1.47)

In the univariate logistic regression analysis, whether FEDN SZ patients had SI was taken as the dependent variable, and the variables with statistically significant differences in **Table 2** were also included as independent variables, revealing the following variables independently associated with SI: TC (*OR* = 2.08, *P* < 0.001), LDL (*OR* = 2.29, *P* < 0.001), positive symptom factor (*OR* = 1.11, *P* < 0.001), depressive symptom factor (*OR* = 1.33, *P* < 0.001). This indicates that when TC levels, LDL levels, the score of the positive symptom factor, and the score of the depressive symptom factor increase respectively, the possibility of SZ patients developing SI is also higher.

Finally, as shown in **Table 4**, s stepwise logistic regression analysis, by dynamically adjusting the model through the gradual introduction of variables, we ultimately retained three factors: TC (*OR* = 5.81, *P* < 0.01), the positive symptom factor (*OR* = 0.81, *P* < 0.05), and the depressive symptom factor (*OR* = 1.30, *P* < 0.001). This indicates that the higher the TC level, positive symptom factor score, and depressive symptom factor score, the higher the likelihood of SI in SZ patients. In this model, the positive symptom factor was masked due to collinearity with serum TC level and depressive symptom factor. The stepwise logistic regression method, by introducing variables in each round, revealed the independent information content of the positive symptom factor, suggesting that it may still have clinical significance. Serum TC levels remained stable under both strategies, further supporting its potential feasibility as a biomarker for suicidal ideation in FEDN SZ patients. However, due to the complexity of suicidal ideation formation and the limitations of cross-sectional studies, the role of serum TC levels in the development of suicidal ideation among SZ patients still requires more in-depth investigation.

**Table 4:** Stepwise logistic regression analysis.

Steps	Variables	Stepwise logistic regression analysis			
		*β*	*S. E*	*P*	*OR (95%CI)*
Step 1^a^	Depressive symptoms	0.28	0.06	<0.001^***^	1.33 (1.18 ~ 1.49)
Step 2^b^	TC	0.66	0.19	<0.001^***^	1.93 (1.34 ~ 2.79)
	Depressive symptoms	0.26	0.06	<0.001^***^	1.29(1.152 ~ 1.45)
Step 3^c^	TC	1.76	0.57	0.002^**^	5.81 (1.91 ~ 17.72)
	Positive symptoms	-0.21	0.10	<0.034^*^	0.81 (0.67 ~ 0.98)
	Depressive symptoms	0.26	0.06	<0.001^***^	1.30 (1.15 ~ 1.47)

Comment: The bold values indicate statistical significance, with *p* < 0.05 denoting significance, *p < 0.05 indicating a significant result, **p < 0.01 representing a highly significant result, and ***p < 0.001 signifying an extremely significant outcome.

a The variable entered in step 1: depressive symptom factor.

b The variable entered in step 2: TC.

c The variable entered in step 3: positive symptom factor.

#### 3.3.2 ROC curve

**Figure 1:**
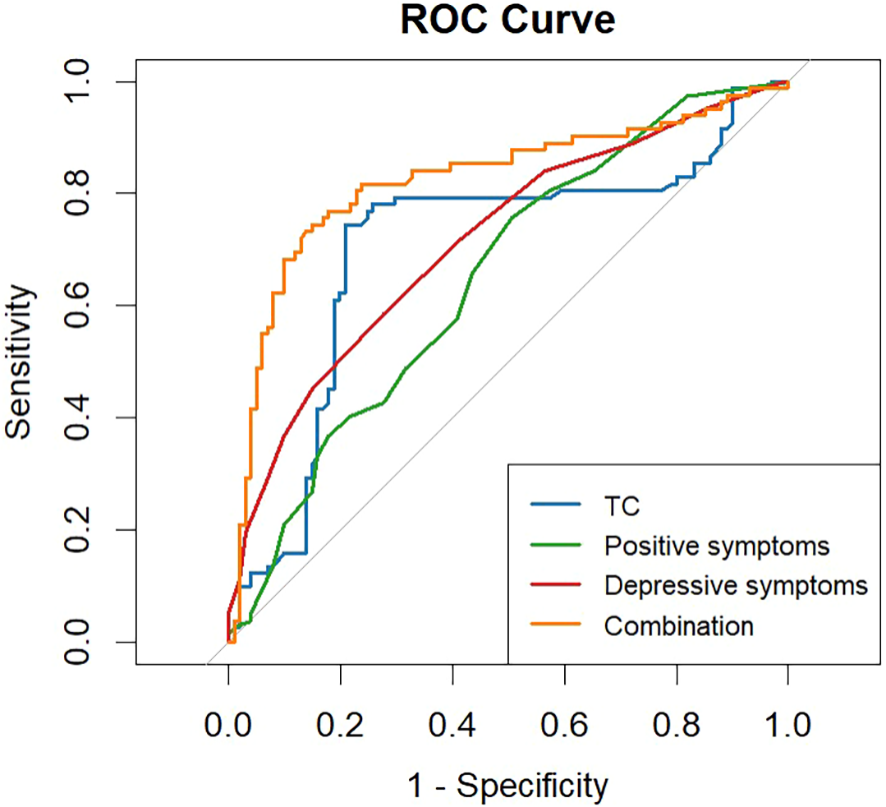
ROC curve illustrating risk factors for SI in patients with SZ. The areas under the four curves in the graph represent the predicted probabilities of SI in SZ patients. Higher values indicate a greater risk of SI. As shown in the figure, the areas under the curve for serum TC levels, positive symptom factor, depressive symptom factor, and their combination are 0.702, 0.650, 0.717, and 0.824.

Through binary logistic regression analysis, we utilized the three independent predictors mentioned above to construct an AUC-ROC curve for predicting the risk of SI in patients with SZ (see **Figure 1**). The AUC-ROC results indicated that the positive symptom factor had the lowest discriminatory ability, with a value of 0.650, while the serum TC level and the depressive symptom factor showed better discriminative power, with values of 0.702 and 0.717, respectively. When all risk factors were combined in the regression model, the AUC-ROC value increased to 0.824, suggesting that the combined factors had a superior ability to distinguish SZ patients with or without SI.

## 4 Discussion

This study investigated the relationship between blood lipid metabolism and SI in patients with FEDN SZ. The findings indicate that (1): Compared with healthy controls, FEDN SZ patients exhibited higher serum levels of TC, TG, and LDL, and lower HDL levels (2); The incidence of SI in patients with FEDN SZ was 44.81% (3); Compared to SZ patients without SI, those with SI show significantly elevated levels of serum TC, HDL, and LDL, alongside significantly higher scores for both positive symptom factors and depression symptom factors (4); In patients with FEDN SZ, serum TC levels, positive symptom factor scores, and depression symptom factor scores are independently associated with the emergence of SI.

This study found that compared with HC subjects, FEDN SZ patients had higher levels of serum TC, TG and LDL, while the level of HDL was lower, which was consistent with the results of previous studies (23), indicating abnormal lipid levels in SZ patients. A meta-analysis indicated that approximately 40% of individuals with SZ and related disorders experience abnormal lipid metabolism (7). Furthermore, studies have indicated that serum TC, HDL, and LDL levels in FEDN SZ patients are lower than those in the HC group, while serum TG levels are higher than those in the HC group (24). Another meta-analysis found that both TC and LDL levels were significantly reduced in first-episode schizophrenia patients compared to the HC group, with a notable increase in TG levels, while HDL levels did not show a significant difference (25). The discrepancies between these studies and our statistical results may be attributed to variations in sample selection. This underscores the prevalence of abnormal lipid metabolism in SZ patients, particularly those experiencing their first episode and not receiving medication, who often exhibit a distinct pattern of lipid metabolism abnormalities. This suggests that such dysregulations in lipid metabolism may stem from the intrinsic pathophysiological characteristics of SZ patients rather than solely from pharmacological treatments (26, 27).

In our study, nearly half (44.81%) of patients with FEDN SZ reported experiencing SI. This aligns with a trend observed internationally. For example, an Indian study pegged the rate of SI in first-episode schizophrenia patients at a substantial 37.25% (28). An Italian study reported that 40.8% of first-episode SZ patients exhibited SI (29). Furthermore, Bai et al. conducted a meta-analysis that revealed a global incidence of SI in SZ patients to be approximately 34.5% (30). It is important to note that variations in the definitions of SZ and differences in sample selection across studies may contribute to discrepancies in their findings.

Our study found that patients with SZ who exhibited SI had higher serum TC levels compared to those without SI. Similar findings have been reported by other researchers; for instance, one study indicated that elevated serum TC levels were associated with SI in women with first-episode SZ (17). Research has indicated that oxidative stress in SZ patients affects the expression of antioxidant genes by influencing various transcription factors, particularly Nrf2. Under oxidative stress, Nrf2 can translocate into the nucleus, enhancing the expression of antioxidant genes, which in turn induces the expression of lipid metabolism genes. Concurrently, oxidative stress affects cysteine residues in signaling proteins through redox reactions. These redox reactions can alter the activity of signaling proteins, thereby inducing changes in intracellular signaling pathways, which subsequently affect the expression of lipid metabolism-related genes, leading to elevated serum TC levels and other alterations. These changes may increase the risk of developing SI (31). However, it is important to note that the relationship between serum TC levels and SI in patients with SZ is not always consistent. According to certain research, reduced TC may correlate with suicidality in schizophrenic individuals (18, 32), particularly among those who have attempted suicide (33). We found similar results in other mental illnesses. Choi et al. found depressed patients with low TC exhibit higher risk of suicidal behavior (34). A different observational study dug into the connection between blood lipid levels and attempted suicide among individuals grappling with obsessive-compulsive disorder. The findings indicated that those with suicidal inclinations tended to have notably lower TC levels compared to their counterparts who didn’t exhibit these tendencies (35). Additionally, Bjanka et al. reported that serum TC levels in male patients with bipolar disorder who had attempted suicide were significantly reduced (36). The observed differences in correlation may be attributable to variations in medication use and the course of the disease.

We found that SZ patients with SI had higher serum HDL levels compared to those without SI. Our results contradict some previous studies; for instance, data from healthy adults in the United States indicated that low HDL levels were significantly associated with attempted suicide in women (37). Additionally, a study by Andrea et al. suggested that a decrease in HDL may be a hallmark of increased suicidal tendencies in patients with obsessive-compulsive disorder (35). It is worth noting that some studies suggest that HDL levels may be associated with different subgroups or symptom dimensions of mental disorders. For instance, a decrease in HDL levels has been observed in patients with treatment-resistant SZ, which may suggest that abnormal lipid metabolism is related to the severity of the disease or treatment response (38). In addition, HDL levels have been found to predict the improvement of depressive symptoms in female patients with major depressive disorder, and higher levels of HDL are associated with better symptom improvement (39). Although these findings come from different groups of mental illness, they all suggest that HDL may play a certain role in the psychopathological mechanism. Concurrently, we observed that SZ patients with SI exhibited higher serum LDL levels than those without SI. This finding also contrasts with previous research. For example, Kavoor et al. reported a significant negative correlation between LDL levels and suicide ratings in SZ patients compared to healthy individuals (40). Furthermore, an earlier SZ study indicated that lower LDL levels were significantly associated with suicidal behavior in these patients (OR = 0.99, 95% CI = 0.98–1.00) (41). In our study, further binary logistic analysis did not reveal a significant correlation between serum HDL and LDL levels and SI. Therefore, while our findings differ from those of previous studies, they underscore the need for further research to elucidate the relationship between serum HDL and LDL levels and SI in SZ patients.

In addition, we found that in patients with SZ, the positive symptom factor score and the depression symptom factor score are independently associated with the generation of SI. Previous studies have also identified a correlation between the negative symptom factors of PANSS, depression symptom factors, and SI in SZ patients. For instance, a study conducted in India indicated that positive symptoms and depression serve as significant predictors of SI in SZ patients (42). Furthermore, research has demonstrated that patients exhibiting psychotic symptoms are at a 2.39-fold increased risk of experiencing SI and a 3.20-fold increased risk of engaging in self-harm behavior compared to those without psychotic symptoms (43, 44). Additionally, Ayesa et al. found that depression symptoms in 383 first-episode SZ patients were significantly correlated with their suicidal behavior (OR = 1.15, 95% CI = 1.06–1.24) (41). These findings suggest that SZ may indeed be influenced by its positive symptoms and depressive symptoms.

Collectively, these findings indicate an intrinsic relationship between elevated serum TC levels and the emergence of SI, potentially attributable to increased oxidative stress (31). Furthermore, our analysis revealed that in patients with FEDN SZ, serum TC levels, positive symptom factor scores, and depression symptom factor scores were independently associated with the development of SI. Additionally, the mechanistic relationship between serum HDL and LDL levels and the onset of SI in SZ patients warrants further investigation.

This study has several limitations. First, it is a cross-sectional study; future research could incorporate longitudinal follow-up to examine the relationship between changes in blood lipids and SI in patients with schizophrenia over different time periods. Secondly, the sample size in this study is relatively small, and future studies should aim to increase the sample size to further develop the model and validate the findings of this research.

## Data Availability

The raw data supporting the conclusions of this article will be made available by the authors, without undue reservation.
